# Cytokine-Mediated Neuroinflammation in Cancer-Related Cognitive Impairment: Mechanistic Insights and Therapeutic Implications for Psycho-Oncology Practice

**DOI:** 10.1192/j.eurpsy.2026.10660

**Published:** 2026-07-31

**Authors:** A. H. I. Abu Shehab, Ș. L. Burlea, A. Lucreția, I. Chiscop, A. A. Ș. Baltă, A. Bulgaru Iliescu, L. Stoica, A. Ciubară

**Affiliations:** 1 Psychiatry, “Elisabeta Doamna” Psychiatry Hospital, Galați, Galati; 2 Psychiatry, “Grigore T. Popa” University of Medicine and Pharmacy (UMF), Iași, Iasi; 3 Internal Medicine; 4 Oral & Maxillofacial Surgery, “Sf. Apostol Andrei” Emergency Clinical County Hospital, Galați; Faculty of Medicine & Pharmacy, “Dunărea de Jos” University of Galați; 5 Medical/Clinical Department & Doctoral School of Biomedical Sciences, Faculty of Medicine and Pharmacy, “Dunărea de Jos” University of Galați, Galati; 6 Morphofunctional Sciences, County Emergency Clinical Hospital “St. Spiridon” Iași; “Grigore T. Popa” University of Medicine and Pharmacy (UMF), Iași, Iasi; 7 Psycho-oncology, Psycho-oncology Clinic Lidia Stoica, Bucharest; 8 Psychiatry, Faculty of Medicine and Pharmacy, “Dunărea de Jos” University of Galați; “Elisabeta Doamna” Psychiatry Hospital, Galați, Galati, Romania

## Abstract

**Introduction:**

Cancer-related cognitive impairment (CRCI) affects up to 75% of cancer patients, significantly impacting quality of life and functional capacity. While traditionally attributed to chemotherapy neurotoxicity, emerging evidence implicates cytokine-mediated neuroinflammation as a central pathophysiological mechanism. The bidirectional communication between peripheral inflammatory processes and central nervous system dysfunction represents a paradigm shift in understanding CRCI etiology.

**Objectives:**

To synthesize current evidence on cytokine-mediated neuroinflammation in CRCI pathogenesis and evaluate therapeutic implications for psycho-oncology practice, focusing on mechanistic pathways and evidence-based interventions targeting inflammatory processes.

**Methods:**

Comprehensive literature review of peer-reviewed publications (2015-2025) examining cytokine involvement in CRCI. Search strategies included PubMed, PMC, and academic databases using terms: “cytokine neuroinflammation,” “cancer cognitive impairment,” “psycho-oncology interventions.” Primary focus on mechanistic studies, clinical trials, and therapeutic intervention research.

**Results:**

Key findings demonstrate that pro-inflammatory cytokines (IL-1β, IL-6, TNF-α) disrupt blood-brain barrier integrity, activate microglia and astrocytes, and alter neurotransmitter systems. Studies showed IL-1β associated with poorer cognitive performance (p=0.023), while IL-6 correlated with perceived cognitive disturbances (p=0.001). Conversely, IL-4 demonstrated neuroprotective effects. Animal models confirm systemic inflammation induces widespread neuroinflammation in hippocampus, cerebellum, and cortical regions. Therapeutic interventions show promise: mindfulness-based stress reduction effectively improves subjective cognitive function, while psychological interventions directly reduce inflammatory markers through HPA axis modulation.

**Conclusions:**

Cytokine-mediated neuroinflammation represents a critical mechanism in CRCI pathogenesis, offering novel therapeutic targets. Psycho-oncological interventions demonstrate dual benefits by addressing psychological distress and modulating underlying inflammatory processes. Integration of mechanistic understanding with evidence-based psychological interventions provides a foundation for personalized, biologically-informed psycho-oncology practice targeting neuroinflammation to improve cognitive outcomes in cancer survivors.

**Disclosure of Interest:**

None Declared

